# Once HIV Knowledge Is Addressed: HIV-Stigma From the Perspective of Healthcare Professionals Working in HIV Facilities

**DOI:** 10.3389/ijph.2026.1609379

**Published:** 2026-03-27

**Authors:** Clara Le Saux, Ingrid Gilles, David Jackson-Perry, Ellen Cart-Richter, Olivier Nawej Tshikung, Katharine E. A. Darling

**Affiliations:** 1 Department of Epidemiology and Health Systems, University Center of General Medicine and Public Health, Lausanne, Switzerland; 2 Human Ressources Department, Lausanne University Hospital (CHUV), Lausanne, Switzerland; 3 Infectious Diseases Service, Lausanne University Hospital (CHUV), Lausanne, Switzerland; 4 Department of Infectious Diseases, Geneva University Hospital, Geneva, Switzerland; 5 Faculty of Biology and Medicine, University of Lausanne, Lausanne, Switzerland

**Keywords:** healthcare professionals, HIV, HIV-stigma, qualitative methods, Switzerland

## Abstract

**Objectives:**

Stigmatising behaviour towards people with HIV (PWH) by healthcare professionals (HCPs) are often linked to poor HIV knowledge. This qualitative study explores how HIV-related stigma affects daily practice when HIV knowledge is high.

**Methods:**

HCPs from HIV care facilities in French-speaking Switzerland–administrative staff, nurses, and physicians–were invited to be interviewed by a team trained in qualitative methods using semi-structured guides. Interview transcripts were analysed with IRaMuTeQ software.

**Results:**

Ten interviews were completed before data saturation was reached. Three themes emerged: 1) clinic reception, 2) care provision for PWH, and 3) HIV knowledge. Administrative staff described challenges in maintaining patient anonymity. These included not greeting people by name and organising appointment schedules so people from shared social groups never meet at the clinic, thus avoiding HIV-status-sharing by inference. Physicians described underestimating stigma experienced by PWH and cited time constraints during consultations to address this. All groups felt that stigma persists due to limited HIV knowledge among the general public and non-specialist HCPs.

**Conclusion:**

Even with good HIV knowledge, HIV-stigma impacts HCP practice and care provision. Efforts to protect anonymity may unintentionally reinforce rather than address HIV-stigma. While improving public and HCP HIV knowledge reduces enacted HIV-stigma, collaborative interventions between HCP sectors and with PWH could help to adapt HCP practices.

## Introduction

Antiretroviral therapy (ART) has changed HIV from a fatal infection to a chronic condition with an excellent life expectancy. Despite medical advances in HIV care, the stigma associated with HIV remains. HIV-stigma impacts on the mental, social and physical health of people with HIV (PWH) and on prevention and treatment outcomes such as HIV testing and ART adherence [1, 2]. Stigma occurs at individual and societal levels and can be considered in terms of drivers (at an individual level), facilitators (at a societal level) and manifestations [3–5]. Stigma drivers include poor HIV knowledge, fear, and conscious or unconscious prejudice towards social groups classically at greater risk of HIV acquisition [5]. Facilitators include healthcare policies or laws. Manifestations are the consequences of drivers and facilitators and lead to stigma constructs including enacted (experienced), anticipated and internalised stigma [2].

Several reviews have examined HIV-stigma experienced by PWH in healthcare facilities [6–8] and interventions to address this [5, 6, 9]. Despite the use of non-consensus study questionnaires [10], the conclusions have been similar, that HIV-stigma could be reduced with HCP education and training regarding, for example, how HIV is and is not transmitted, how to care for different patient populations, and how to optimise local policies or physical space [6]. These reviews cite mostly quantitative studies examining HCPs working outside the field of HIV.

We conducted a qualitative study of HIV-stigma among HCPs working in the field of HIV in Switzerland. We chose this population to examine how HIV-stigma might manifest in facilities where HIV knowledge is good. The study objectives were to determine the impact of HIV-stigma on HCP daily practice, how HCPs talk about stigma with PWH and how HIV-stigma affects patient care.

## Methods

### Patient and Public Involvement

People living with HIV were involved throughout the study process, from submitting the study protocol for ethical approval, to interview design and manuscript review.

### Study Setting

HCPs (participants) were recruited from three professional groups: 1) administrative staff and 2) nurses (clinic and specialist) at Lausanne University Hospital Infectious Diseases Outpatients Service (LUHIDOS), and 3) infectious diseases physicians in French-speaking Switzerland affiliated to the Swiss HIV Cohort Study (SHCS) [11].

LUHIDOS sees non-HIV infectious diseases outpatient referrals and is the cantonal university hospital centre for the treatment of PWH. Administrative staff at this centre are the first point of contact with patients. They receive telephone enquiries, organise appointments and assign patients to appropriate nurses or physicians. Patients attending appointments who cannot be seen immediately are invited to wait in the clinic waiting area. People in this area may be attending for HIV care or for a non-HIV-related infectious diseases consultation. Clinic nurses provide services of phlebotomy, vaccination, administration of long-acting ART, and intravenous treatment. When PWH attend for their 3- to 6-monthly blood tests, they take a brief social and medical history to identify any problems prior to the medical visit. Specialist nurses provide psychological and social support and see HIV-negative people presenting for pre- or post-exposure HIV prophylaxis. Finally, SHCS-affiliated physicians are all trained in infectious diseases and work in the field of HIV at university hospitals or in private practice.

Prior to this study, LUHIDOS had engaged in several service-level HIV-stigma education seminars, as part of continued professional development in a university hospital setting. It had also led a quantitative pan-Swiss HIV-stigma survey, nested within the SHCS, using a validated 12-item stigma questionnaire [12], during which physicians had spoken about HIV-stigma to 5,563 PWH attending SHCS-affiliated HIV facilities [13]. The current study was the second of a two-step *qualitative* study, examining HIV-stigma from the perspectives of PWH and HCPs. The first step, involving PWH, has been described previously [14].

### HCP Recruitment

The study was presented to HCPs at LUHIDOS and to a study physician (ONT) at Geneva University Hospitals. SHCS-affiliated physicians were invited to participate via the SHCS network. Inclusion criteria were working in a post involving regular contact with PWH and speaking French sufficiently fluently for conversation. The interviews were conducted in French as this is the language spoken in the cantons where Lausanne and Geneva are situated.

HCPs wishing to participate contacted the principal study investigator (KD) who passed each HCP’s contact details to the study team member coordinating and conducting the interviews (IG). This study used an interpretive phenomenological approach to capture perceptions participants have regarding HIV-stigma (what they think and believe) in the social and healthcare context [15]. Consistent with this framework, judgement sampling was applied based on the study objectives, ensuring that at least two individuals from each HCP group were included. Participants were assigned a study code number to maintain anonymity and the study member responsible for recruitment (KD) was different to the member conducting interviews (IG). Recruitment ended once data saturation was reached. This occurs when the analysis of the interviews no longer yields new information and the identified themes are repeated.

### Interviews, Data Collection and Analysis

Interviews were conducted by one female researcher trained in qualitative interview methods (IG) between April and May 2022, either face-to-face (nine participants) or via an online video conferencing platform (one participant), depending on participants’ availability to attend in person. Interviews were designed to last 45–60 min, and a token remuneration of 20 Swiss Francs (approximately 19 Euros) was offered (albeit declined by several participants).

An interview guide was developed based on the literature on HIV-stigma in healthcare settings available at the time of the study and through patient and public involvement. Themes included public and non-HIV-specialist HCP perceptions of PWH, impact of HIV-stigma on health and the social and professional lives of PWH, HCP perceptions of HIV-stigma, and experiences of discussing HIV-stigma with PWH. The guide was adapted by HCP group when necessary, so that topics covered corresponded to each participant’s daily practice. Interviews were audio-recorded and transcribed. Audio-recordings were destroyed following transcription.

Interview transcripts were analysed using IRaMuTeQ lexicographic computer-assisted qualitative data software (version 0.7 alpha2, 2008-2014, Pierre Ratinaud) which has been used to analyse qualitative data in the context of HIV and other health domains [14, 16, 17]. Using the Reinert method [18], the software classified every part of the text based on the co-occurrences of words or expressions used, as described elsewhere [14]. This classification provided main themes structuring participants’ discourse, each with its own vocabulary and specific text segments. These themes were then reviewed by a separate member of the study team (CL) and cross-referenced with the transcripts. Discussion sessions were held within the study team to ensure that theme interpretations agreed with interview content. Once theme meanings were identified, strengths of association between vocabulary and themes were examined.

## Results

Data saturation was reached after interviews with ten HCPs: two administrative staff, two clinic nurses, two specialist nurses, and four physicians. Of the physicians, two were working at LUHIDOS, one at the equivalent university hospital unit in Geneva and one in private practice in Lausanne (external to LUHIDOS). Since interview analyses revealed no differences between clinic and specialist nurses, nurses were analysed together as a single professional group.

**FIGURE 1:**
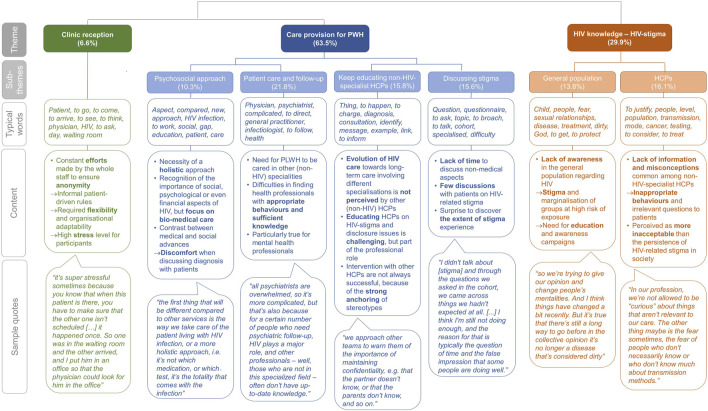
Themes, sub-themes, typical words, content, and sample quotes derived from interview transcripts. The questions asked in the cohort in the “Discussing stigma” sub-theme is referring to a quantitative study on HIV-stigma based on a 12-item questionnaire among people living with HIV enrolled in the Swiss HIV cohort study which was performed prior to the current study [13]. Abbreviations: PWH, people with HIV; HCPs, healthcare professionals (Lausanne, Switzerland, 2020).

Computer-assisted analysis identified three main themes: 1) clinic reception, 2) care provision for PWH, and 3) HIV knowledge, the latter two being sub-divided into six sub-themes. These themes represented respectively 6.6%, 63.5% and 29.9% of the analysed text (Figure 1). Themes, sub-themes, typical words, content and sample quotes are presented in Figure 1. Additional quotes, illustrating specific aspects of the themes and sub-themes, are presented in Supplementary Material.

### Clinic Reception

This theme (6.6% of the analysed text) was characterised by vocabulary related to the waiting room and patients’ requests and wishes (Figure 1). Discourses related to clinic reception were prominent among administrative staff. They described a constant effort to maintain patient anonymity, as people attending for HIV treatment had historically explicitly requested non-named identification through fear of inadvertent HIV status disclosure. This fear was so strong that some PWH avoided the waiting area. This created an impersonal atmosphere in the clinic and implicit rules between the staff and PWH. One participant explained she had to know patients well enough to not call them by name, but instead to go to them directly when it was their turn to be seen. The regular turnover of junior physicians presented a challenge, as administrative staff had to ensure new colleagues were aware they should not call patients by name. This patient-driven choice raised the importance of organisational adaptability and of a flexible approach to ensure different preferences were accommodated within the service. In addition to knowing PWH well enough to not call them by name, it was also necessary to know which people were part of the same social circle, so as to not schedule appointments on the same date and avoid inadvertently inferring that a person was living with HIV. This was especially true for PWH of non-Swiss origin. The challenge of meeting individual demands and the risk of inadvertently overlooking or failing to meet individual preferences added stress to an already demanding job.

### Care Provision for PWH

This main theme was divided in four sub-themes. This was the largest theme, representing 63.5% of the analysed text (Figure 1).

#### Psychosocial Approach

Participants described a psychosocial approach as being a critical aspect of HIV care, requiring a “*holistic*” understanding of PWH. They described how living with HIV has an influence not only on physical health, but on all aspects of life, with social, psychological, and even financial stakes. Whilst they recognized these non-medical aspects, they acknowledged they tended to concentrate on providing medical care which they considered their core role. For example, even if physicians agreed that helping PWH in dealing with HIV-stigma was important, they described not having sufficient time to do this. Physicians were regarded by other HCP groups as not being well-placed to discuss stigma, due to time constraints and their role as medical providers. In this context, interdisciplinary collaboration was considered vital. While nurses regarded PWH with undetectable viraemia as medically well, they would see patients outside medical consultations to review psychosocial wellbeing.

The contrast between medical advances in HIV and the challenges still faced by PWH in their daily lives because of stigma was striking for participants. When giving an HIV diagnosis, participants wanted to be positive as the medical news for PWH is good. However, they emphasised that dealing with the social aspects of HIV remains difficult.

#### Patient Care and Follow-Up

Participants felt they were well-trained to support PWH in their medical HIV journey but less well-equipped to help deal with stigma. They highlighted the need for patients to be cared for in other (non-HIV) specialities, particularly for psychological support, and difficulties in finding HCPs with sufficient HIV knowledge and experience. Although they had lists of potential HCPs, these did not meet patients’ needs. They also described general practitioner (GP) shortages and lack of HIV knowledge among GPs, which was problematic for continuity of care. They described the challenge of patient retention in care and in finding a balance between offering support and ‘harassing’ PWH who miss clinic appointments. Finally, participants described difficulties in maintaining high quality follow-up in a university hospital setting due to the frequent junior physician turnover. Regarding HIV-stigma, participants felt that a long-term approach was required to create a relationship of trust with patients and to support them throughout their HIV journey.

#### The Need to Keep Educating Non-HIV-Specialist HCPs

Participants observed that their non-HIV-specialist colleagues did not always understand that, as HIV as a condition has evolved, living with HIV has also changed and that HIV is now a chronic disease in which long-term continuity of care is essential.

Educating non-HIV-specialist HCPs regarding HIV-stigma and HIV-status confidentiality was a challenge. Participants actively tried to raise awareness about maintaining confidentiality, especially when PWH had not disclosed their HIV status. However, they encountered judgement from colleagues, for example, for withholding a person’s HIV status from a partner or family. Participants sometimes faced negative reactions when attempting to make their colleagues aware of the individual and interpersonal challenges associated with living with HIV. Participants considered that educating peers about living with HIV was part of their professional role. However, they recognised that such interventions were not always successful. The social perceptions and stereotypes associated with living with HIV were deeply rooted and difficult to deconstruct, even among fellow HCPs.

#### Discussing Stigma

Participants explained that discussions during clinic visits were often focused on medical care, leaving little room to discuss patients’ experiences and challenges. Physicians mentioned time constraints and so non-medical aspects were addressed by nurses rather than during medical consultations. All participating physicians had recruited PWH to a previous SHCS study on HIV-stigma^13^ which required them to complete a stigma survey with the PWH they followed in clinic. The physicians admitted that, prior to that study, few of them had discussed stigma with their patients. For the few physicians who had discussed HIV-stigma out with the SHCS study, this was not routine practice. Stigma was discussed, for example, when patients brough the subject up themselves or showed visible signs of distress. Some physicians stated that, before working with PWH and before the SHCS study, they did not consider stigma a concern. They also felt insufficiently trained to introduce stigma conversations. After discussing stigma with patients, they were surprised to discover how frequently and silently PWH experienced stigma. They realised they had work under the false impression that the patients they saw in clinic did not experience stigma because they did not talk about it.

### HIV Knowledge–HIV-Stigma

This theme represented 29.9% of the analysed text and was defined by terms connected to misconception, fear, and education (Figure 1). The theme was divided into two sub-themes.

#### General Population

Participants described a lack of awareness in the general population regarding HIV in the ART era, and a widespread fear of death and misconceptions about modes of transmission. The participants felt this lack of awareness fuelled HIV-stigma. They believed groups at risk of HIV acquisition were highly stereotyped, perpetuating harmful generalisations and further marginalising PWH and all people belonging to stereotyped groups, whether living with HIV or not. Participants observed that false representations of HIV persist throughout society and described a critical need for awareness campaigns and education to address misconceptions, disseminate accurate information and tackle stigma.

#### HCPs

Participants also described misconceptions among non-HIV-specialist HCPs and inappropriate behaviour (enacted stigma), including irrelevant questions during patient interactions. Commenting on a specific instance of inappropriate behaviour, one participant expressed the idea that these behaviours arose from ‘*unfounded fears’* and *‘ignorance’* about current HIV care. Participants cited unnecessary infection control precautions when treating PWH. One recounted being contacted by a surgeon who was worried about operating on a PWH with undetectable viremia. Despite time spent explaining the absence of HIV acquisition risk in this case, the participant was disappointed the surgeon had ended the discussion insisting she would still wear two pairs of gloves. Behaviour based on misconceptions among HCPs was perceived as more unacceptable than HIV-stigma in society and highlighted a need for further HCP education and training.

## Discussion

In this qualitative study of HCPs working with PWH in Switzerland, we have observed that, even in a healthcare facility in a resource-rich country where HIV knowledge is good, HIV-stigma remains a challenge in daily practice. Clinic reception represented only 6.6% of the discourse but highlighted significant organisational challenges, whereas care provision (63.5% and HIV knowledge/HIV stigma (29.9%) accounted for the majority of text segments. Administrative staff described a complex workload generated by maintaining different approaches to patient confidentiality. Maintaining secrecy and the fear of inadvertently inferring an individual’s positive HIV status created additional work pressures. HCPs emphasised the holistic and long-term approach necessary in HIV care and how this may be impacted by frequent staff turnover, but also by the HIV-stigma experienced during interactions with non-HIV-specialist HCPs. After taking part in a previous HIV-stigma survey, physicians recognised that they had underestimated the stigma which PWH experience. They described insufficient time during medical consultations to both fulfil their medical role and discuss stigma with patients. However, they also recognised they felt insufficiently trained to discuss non-medical aspects of HIV with PWH. Finally, all three HCP groups identified lack of HIV knowledge in the general population and among non-HIV-specialist HCPs as factors perpetuating HIV-stigma.

The majority of reviews on HIV-stigma in healthcare settings cite quantitative studies conducted in the United States or in low- to medium-income settings [6, 7]. The literature on HIV-stigma among HCPs in Europe has been sparse, although a recent large report quantified HIV knowledge among HCPs [19]. Our study is novel in focusing on HCPs based solely in HIV facilities and in using qualitative methods. We identified two studies similar to our own. In the Netherlands, Stutterheim et al conducted a mixed methods study in 2014 which included semi-structured interviews with 14 non-HIV-specialist HCPs [20]. These HCPs reported having only basic HIV knowledge and little experience with PWH. Whilst they felt that HIV was ‘normalised’ and no longer stigmatised, they reported practices at odds with this stance. Such practices, driven by fear of occupational exposure, included additional infectious control precautions when treating PWH, and labelling patients’ medical files to ‘warn’ other HCPs. They also reported curiosity among colleagues who speculated about modes of patients’ HIV acquisition [20]. More recently (2023), Moseholm et al conducted a survey among HCPs in infectious disease and obstetrics and gynaecology services at a Danish university hospital using the ‘Measuring HIV stigma and discrimination among health facility staff’ questionnaire [21, 22]. Compared to infectious diseases specialists, obstetrics and gynaecology HCPs had less HIV training and, although their level of fear and negative attitudes towards PWH was low, they were more likely to use additional infection control precautions [22]. Both these studies suggest a discrepancy between HCP attitudes to PWH and their clinical practice, with unnecessary precautions taken by HCPs with less HIV experience. The HCPs in our study also observed misconceptions and ‘unfounded fears’ among their non-specialist colleagues. What our study adds to the literature is that, despite the advances in medical aspects of HIV and despite the awareness that discussing HIV-stigma is part of care, some HCPs, even those working in HIV facilities, felt insufficiently trained to discuss HIV-stigma with their patients. This feeling was prominent among physicians and stemmed from their experience of completing a validated HIV-stigma questionnaire for a previous, quantitative, HIV-stigma study. In completing the questionnaire, many physicians discussed HIV-stigma with their patients for the first time, and some found they had underestimated stigma as a problem. Having a standardised ‘template’ of questions to broach the topic of HIV-stigma would be one way to open a stigma dialogue between HCPs and PWH. Whilst this might require an initial investment of time, a stigma dialogue would create a channel for HIV-stigma to be discussed and then addressed at subsequent clinic visits.

By not having a stigma dialogue, HCPs implemented counter-productive adjustment practices such as hiding patients, training new staff to not call out patients’ names, and organising appointment schedules so that patients who know each other do not meet in the waiting area. While these practices, highlighted particularly by administrative staff, were derived from patients who were fearful that their HIV status would be revealed by being seen in the clinic, they were constraining and stressful for HCPs, and arguably went beyond simply maintaining confidentiality. Making space for discussing stigma would 1) optimise communication between administrative staff and clinical HCPs to heighten awareness among the latter group of internalised and anticipated stigma experienced by their patients and 2) promote a dialogue between HCPs and PWH to examine how to address stigma both in healthcare settings and in general.

HCPs are undoubtedly invested in the wellbeing of patients and, in the context of HIV, a highly stigmatised condition, this includes protecting PWH from discrimination. The participants described enacted stigma they had observed in non-HIV healthcare settings and described difficulties in finding HCPs with HIV knowledge and experience. One phenomenon related to this, but not directly described by the participants in this study, is the- practice of suggesting to people newly diagnosed with HIV that they do not share their HIV-positive status, to protect themselves from enacted stigma. This phenomenon was encountered in our earlier study on HIV-stigma from the perspective of PWH [14]. Whilst non-status-sharing may protect PWH in the short term, this should be reviewed throughout a person’s HIV journey [14]. PWH participating in our earlier study highlighted that advice from HCPs to not share their HIV-positive status could 1) be perceived as an unquestioned recommendation and 2) delay acceptance of living with HIV [14]. Moreover, the secrecy through non-sharing may become an additional burden to the burden of living with HIV [14]. There is accumulating evidence that HIV-status sharing reduces the stress of anticipated stigma, improves wellbeing, and increases social support, coping and health promoting behaviours including ART adherence [23–25]. Education sessions and structured stigma reduction interventions can support HIV-status sharing appropriate to the individual [26]. The results of the current study suggest that efforts should be invested in the training of HCPs, including administrative staff [6], in caring for PWH, so that non-HIV HCPs have better HIV knowledge and that HIV HCPs discuss HIV-stigma among themselves and with the PWH they care for.

Another finding in our study was that HCPs considered that HIV care is no longer purely medical but must be holistic. This is consistent with the Health Stigma and Discrimination Framework proposed by Stangle et al. which states that to understand stigma phenomena and conduct appropriate interventions, it is necessary to consider stigma at different levels (individual, interpersonal, institutional, community and public policy levels). Considering the constraining measures described by our participants to preserve confidentiality in the waiting area, HIV-stigma affects PWH, HCPs and, more globally, institutional functioning. Within the Health Stigma and Discrimination Framework, interventions focusing on all levels are required to tackle HIV-stigma in healthcare settings, in addition to training aimed at improving HCP HIV knowledge. Whilst the HCP participants in our study expressed willingness to support PWH, HIV-stigma-reduction interventions need to be developed in collaboration with PWH: it is logical that changes to healthcare settings are made with involvement of the people who access these facilities.

### Limitations

This study has limitations. The number of participants was small and may not be representative of HCP views throughout French-speaking Switzerland. However, this was the number at which data saturation was reached. As several participants came from the same centre, our findings may not be generalisable to other settings. Moreover, the fact that multiple participants shared the same organisational context may have contributed to a more homogeneous discourse and so data saturation. Against these limitations, a strength of this study was the inclusion of administrative staff, a group frequently omitted from studies on HIV-stigma among HCPs whilst being the first point of contact with patients attending a facility. Including reception administrative staff in our study shed light on the potential for HIV-stigma in reception and waiting areas which was not described by other HCP groups. Another strength is that all ten interviews were conducted by the same interviewer, limiting variability to study participants.

### Conclusion

In conclusion, we have described how HIV-stigma can impact HCP practice and care provision, even when lack of knowledge, experience and/or resources is removed from the equation. Our observations suggest that reviewing well-intentioned efforts to encourage PWH to avoid sharing their HIV status may help to address rather than reinforce HIV-stigma. Whilst improving public and HCP HIV knowledge reduces enacted HIV-stigma, collaborative interventions at different levels (individuals, institutional, community) could help adapt HCP practices.
