# Supplementary material for: Once HIV Knowledge Is Addressed: HIV-Stigma From the Perspective of Healthcare Professionals Working in HIV Facilities
Source: Int J Public Health. 2026 Mar 27;71:1609379. doi: 10.3389/ijph.2026.1609379 (PMC13065731; doi:10.3389/ijph.2026.1609379)
Supplement: Supplementary file 1 [file Supplementaryfile1.docx]

**Supplementary Material 1: Additional extracts derived from interview transcripts for themes and subthemes (Lausanne, Switzerland, 2020)**

1. **Clinic Reception**

**Typical extracts for the theme *Clinic Reception***

“*Then someone comes along and this person is going to tell you their name very softly because they don’t want us to know […] we know that she comes by saying it softly, she’s afraid to even say her name”*.

“*Some patients really hide. Yes, so the doctor comes to get him, and I tell him it’s your patient sitting outside the office, so he’s not in the waiting room but he’s over there where there are two chairs at the end of the corridor*”.

“*Assistant doctors or new physicians, when they arrive and they don’t know the service, we always tell them: you’re coming to me and you ask me who your patient is and I will show you”*.

“*There are two, and we know we can’t schedule them on the same day, so it’s super-stressful sometimes because we know that when this patient is here, we have to check that the other is not scheduled*”.

1. **Care provision for people living with HIV (PWH)**

**Typical extracts for the sub-theme *Psychosocial approach***

"*A holistic approach to patients living with HIV means it's not just about specific medications or tests; it's about the entirety that comes with this infection, including social support, psychological support, overall education, precautions, and adapting to daily life*”*.*

*“I think I still don't do it enough and the reason is typically that it's the question of time and the false impression that some people are fine. Because they don't say anything, because they hide it. That's my medical point of view. On the other hand, I have the reassurance that there is always, almost always or often, a specialised nurse who, for me, plays a very central role in our unit, and who will very rigorously and systematically address these issues”.*

*“I think we have a rightful place as nurses to be able to work in an interdisciplinary way with patients who are living with it, because it's true that some physicians say he can come in six months, he's undetectable […] but there are a lot of things at play behind this patient and maybe […] we can see him in between to be able to ask certain questions, so it's true that we're very reassured from a medical point of view, but we're less reassured from a psychosocial point of view”.*

"*What is really characteristic is this huge gap between the excellent medical news we give to the patient and then all the difficulties that we are aware people living with HIV will face in their life journey”.*

“*There is the whole aspects [the stigma], of which we are very aware, the patient is probably really aware too, but which is not verbalised from the start”.*

**Typical extracts for the sub-theme *Patient care and follow-up***

“*I think the first thing is to identify the need and orientate if necessary to a psychologist or a psychiatrist; I believe that my role ends here because I am not a psychiatrist*”.

“*For those in need of psychiatric follow-up due to HIV, professionals outside this specialised field [HIV] often lack up-to-date knowledge”*.

“*we have also started to build up a list of professionals with whom we have a good collaboration […] but it is a reality, everyone is overwhelmed, all psychiatrists are overwhelmed, making it more complicated”.*

*“many patients don't have a general practitioner and use the infectious disease physician to wear both hats, and that's complicated, especially as they get older”; “things were set up to have listings of [HIV friendly] psychiatrists or general practitioners or whatever. After that, it’s true that the limits of the system mean that there aren’t necessarily any places available”*.

"*Sometimes we meet once or twice, then schedule another appointment, and the patient doesn't show up. Finding the right balance between being present and not being perceived as 'harassing' is also a challenge. We offer this space, but the person needs to be receptive*”.

**Typical extracts for the sub-theme *The need to keep educating non-HIV-specialist healthcare professionals (HCPs)***

"*Our advantage is that often we stay, sometimes for years. This allows us to follow the person for an extended period, assessing needs, setting objectives, and having intermittent contact. If issues reappear years later, they often come back to us”.*

"*We approach teams to warn them, to make them aware of the importance of maintaining confidentiality, for example, that the partner or parents may not be aware. Yet, we often encounter judgment from caregivers”.*

**Typical extracts for the sub-theme *Discussing stigma***

*“the questions about stigma forced us to open up on these aspects and then there was a bit of a gap between the idea of the patient who seems to be well fulfilled […] [nothing suggested] and that there might in fact be difficulties for [a married person] with children in a well-integrated job”.*

“[I have] *the false impression that some people are fine. Because they don't say anything, because they hide it.”* A participant said that he brought up stigma with *“people who seem […] to be fragile from this point of view, either because they have suffered a lot from stigma or because [he has] the impression that they don’t necessarily say it, but that they experience stigmatisation badly”*.

1. **HIV knowledge – HIV-stigma**

**Typical extracts for the sub-theme *General population***

“*I think it's mainly due to ignorance about it because people don't realise that we don't die from HIV anymore.* [...] *In fact, there are even people who believe that by drinking from the same glass, we can get HIV".*

A participant evoked this by stating *“The term isn't pretty, but dirty, you see, where people have this impression that necessarily, you've got HIV there's something wrong. That you're a bit dirty, you know?”*.

**Typical extracts for the sub-theme *HCPs***

“*There is no objective reason to justify that; on the other hand, it clearly reflects the ignorance even among professionals, including doctors and nurses, regarding up-to-date the care for people living with... For me, it's really a story of ignorance and unfounded fear, but there's never any justification...*”*.*

“*I said, you continue with your usual measures. And then, she answered: but I think that is not enough, I don’t want to get HIV! That was her reaction! […] I had to explain her things that she should know as a graduated physician, not as a specialist or as a surgeon, but as a graduated physician. She is perpetuating the problem*.”
